# Psychosis: Risk Factors and Prognosis

**DOI:** 10.1192/j.eurpsy.2022.797

**Published:** 2022-09-01

**Authors:** M. Fernández Lozano, I. Santos Carrasco, B. Rodríguez Rodríguez, N. Navarro Barriga, M.J. Mateos Sexmero, T. Jiménez Aparicio, C. De Andrés Lobo, C. Vallecillo Adame, M. Queipo De Llano De La Viuda, G. Guerra Valera, A. Gonzaga Ramírez, J. Gonçalves Cerejeira, O. Segurado Martín

**Affiliations:** 1 Hospital Clínico de Valladolid, Psychiatry, Valladolid, Spain; 2 Clinical Hospital of Valladolid, Psychiatry, Valladolid, Spain; 3 Hospital Clínico Universitario, Psiquiatría, Valladolid, Spain; 4 Hospital Clínico Universitario de Valladolid, Psiquiatría, VALLADOLID, Spain; 5 Hospital Clínico Universitario de Valladolid, Psychiatry, Valladolid, Spain

**Keywords:** Psychosis, substance use, risk factor, Childhood Trauma

## Abstract

**Introduction:**

There are life events that may increase the possibilities of suffering some kind of Psychopathology. The most validated model for understanding the aetiology of psychosis is based on genetic and environmental risk factors and their interaction, likely involving epigenetic mechanisms. It is necessary to consider those events as risk factors for Mental Health.

**Objectives:**

Study of risk and prognostic factors in psychosis.

**Methods:**

Review of scientific literature based on a relevant clinical case.

**Results:**

We present the case of a 28-year-old male patient from Peru, currently living in Germany. History of sexual abuse in childhood. He started taking drugs at the age of 8. In the emergency department, he reports that since the beginning of the pandemic, after listening to a speech by the Pope, he begins to interpret signals about situations occurring around him. He begins to read about mystical-religious subjects, changes the style of music he listens to and recognises changes in his personality. He says for months he has been feeling watched, persecuted and expresed someone wants to kill him. He says hears voices and that they communicate with him through bodily sensations.

**Conclusions:**

Childhood trauma, immigration and cannabis use are significantly associated with an increased risk of functional psychosis. A neurotic personality also independently contributes to this risk. The accumulation of these factors increases vulnerability to mental disorders and leads to a worse prognosis and evolution of these pathologies. These findings could help to improve the prevention of psychosis and the development of specific treatment strategies in this particular population.

**Disclosure:**

No significant relationships.

